# Obstructive Sleep Apnea Syndrome: The Effect of Acute and Chronic Responses of Exercise

**DOI:** 10.3389/fmed.2021.806924

**Published:** 2021-12-24

**Authors:** Vasileios T. Stavrou, Kyriaki Astara, Konstantinos N. Tourlakopoulos, Eirini Papayianni, Stylianos Boutlas, George D. Vavougios, Zoe Daniil, Konstantinos I. Gourgoulianis

**Affiliations:** Laboratory of Cardio-Pulmonary Testing and Pulmonary Rehabilitation, Department of Respiratory Medicine, Faculty of Medicine, University of Thessaly, Larissa, Greece

**Keywords:** cardiopulmonary function, metabolism, cognitive decline, physical activity, pulmonary rehabilitation

## Abstract

Obstructive Sleep Apnea Syndrome (OSAS) is a sleep disorder with high prevalence in general population, but alarmingly low in clinicians' differential diagnosis. We reviewed the literature on PubMed and Scopus from June 1980–2021 in order to describe the altered systematic pathophysiologic mechanisms in OSAS patients as well as to propose an exercise program for these patients. Exercise prevents a dysregulation of both daytime and nighttime cardiovascular autonomic function, reduces body weight, halts the onset and progress of insulin resistance, while it ameliorates excessive daytime sleepiness, cognitive decline, and mood disturbances, contributing to an overall greater sleep quality and quality of life.

## Obstructive Sleep Apnea Syndrome

Obstructive Sleep Apnea Syndrome (OSAS) is a disorder of sleep breathing characterized by prolonged periods of complete or partial obstruction of the upper airway (1). OSAS demonstrates increasing prevalence, as it is conjoined with obesity, ranging in 9–37% in men and 4–50% in women, regardless of race and nationality (2). Despite being easily recognized, it tends to elude clinicians' attention, as in only 10% of the population the definitive diagnosis is established (3). Obstructive episodes accompanied by respiratory effort, cause a decrease of the airflow in the upper airway by at least 30% for 10 s and oxygen desaturation in blood by at least 4% (hypopnea) or complete cessation (apnea) for 10 s, resulting in desaturation of oxyhemoglobin and fragmentation of sleep.

The severity of OSAS is evaluated mainly through the Apnea - Hypopnea Index (AHI), representing the number of apneas and hypopneas per hour sleep. Normal values in adults are AHI ≤ 5, 6-to-15 are characterized as mild, 16-to-29 moderate and ≥30 severe OSAS (1). The gold standard for diagnosis and severity evaluation is via polysomnography (PSG). PSG offers a systematic collection of various systematic parameters at the same time during sleep. It utilizes electroencephalogram, electro-oculogram and electromyogram for the discrimination of sleep stages and underlying conditions of the nervous system. In addition, electrocardiogram and pulse oximetry estimate heart rate and rhythm and O_2_ tissue supply, unveiling any disruptions in oxygenation; a hallmark of the pathophysiology of OSAS (4).

Almost 80% of OSAS patients report excessive daytime sleepiness, signifying declining performance at work as well as increased risk for labor and traffic accidents (5). Daytime sleepiness, lack of concentration, fatigue, social and emotional difficulties are likely to cause frictions in relationships with other people and render them susceptible for rather lonely and sedentary lifestyle, as well as for anxiety and depressive disorders (6, 7).

**Figure 1:**
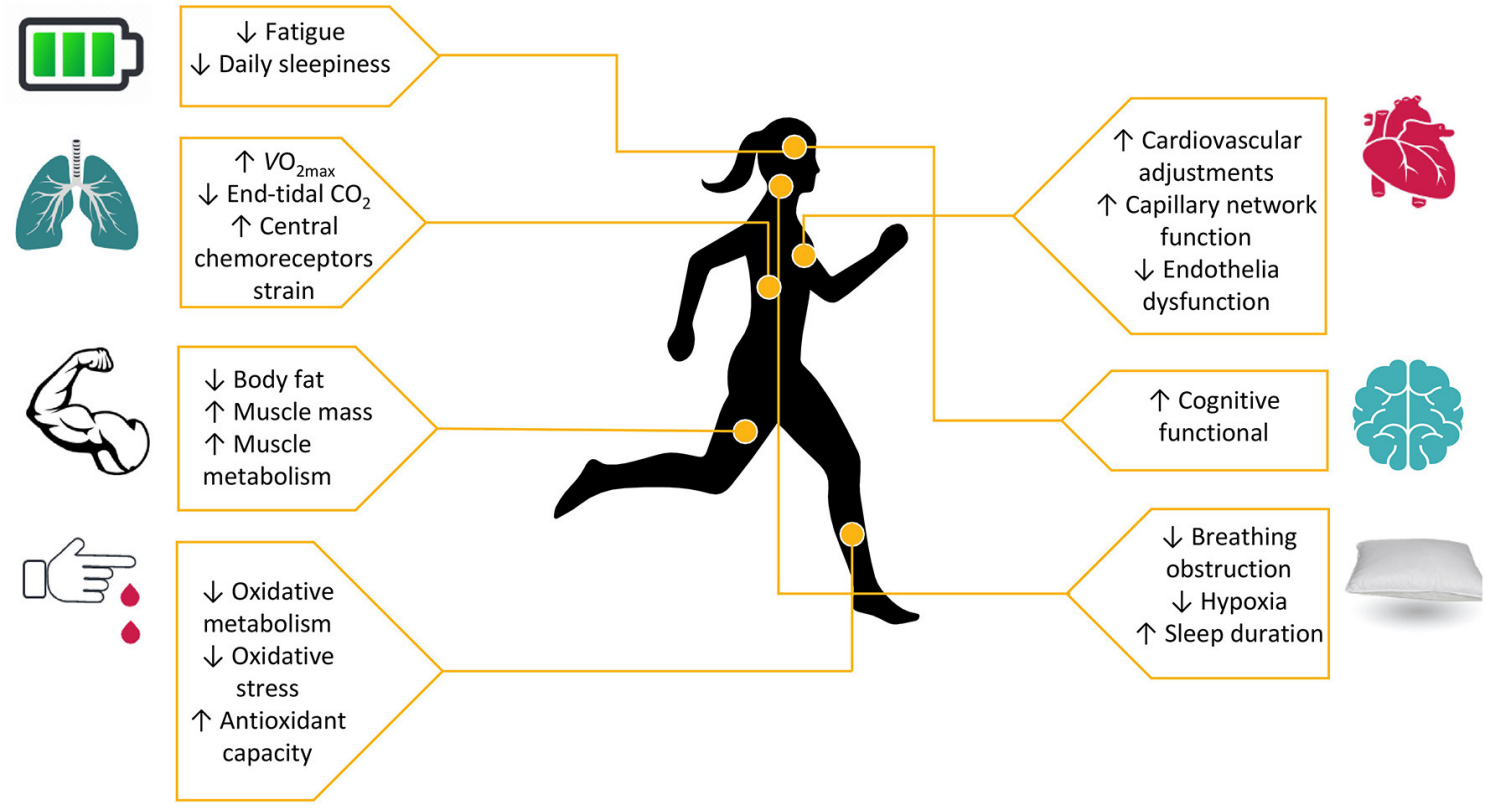
Exercise as a regulation tool to cardiopulmonary, metabolic and cognitive disorders in sleep insufficiency.

Exercise, along with sleep, offers a wide variety of benefits and constitutes a fundamental element for prosperity and longevity. For the general population, it is a necessary constituent of daily life, while in patients, based on their underlying condition, it is crucial to prescribe exercise adjunctively to the main treatment, depending on their capabilities. OSAS patients are faced with crucial systematic consequences, which exercise could ameliorate. They can benefit from exercise, as it improves (Figure 1) cardiopulmonary, cognitive and metabolic profile as well as quality of life, regardless with CPAP therapy and BMI management (8). Oftentimes, CPAP therapy may be insufficient or not tolerated by patients. We added a note to clarify that we examined exercise adjunctively to main therapies, like CPAP, or when such therapies fail or are not tolerated by patients. Therefore, we aimed to review the literature, to describe the acute and chronic systematic detrimental consequences of OSAS with focus on exercise effects, as well as to stress the importance of an exercise program for these patients, adjunctively to main therapy. In Table 1 present a typical exercise program as a strategy to improve health of patients with OSAS.

**Table 1:** Recommended exercise program for patients with OSAS.

**Duration**	**3–9 months**	
Frequency	3–5 session per week	
Session duration	45–60 min	
Warm-up	15% of each session	30–50% of VO_2max_ and/or 50–60% of HR_max_
Aerobic exercises	60% of each session	Intermittent exercise on 70–80% of VO_2max_ and/or on 75–85% of HR_max_
Strength exercise	15% of each session	Multi-joint exercise (large muscle mass), 2–8 sets to 6–12 repetitions on 60–70% of 1 RM
Mobility-Flexibility	10% of each session	Static or dynamic. Stretch to the point of feeling tightness or slight discomfort, 2–4 sets to 6–12 repetitions at 10–30 s
Cool-down	15% of each session	40–50% τ*ης* VO_2max_ and/or 50–60% τ*ης* HR_max_

*1 RM, one-repetition maximum; HR, heart rate; VO_2max_, maximal oxygen uptake*.

## Methods

The choice of literature was done aiming at a comprehensive coverage of the topic during the period January 2019 to July 2021 with keywords: “Obstructive Sleep Apnea Syndrome,” “Sleep disorders,” “Exercise,” “Cogition,” “Oxidative stress,” “Cardiopulmonary Exercise Testing,” “6-minute walk test,” “Fatigue,” “Anxiety,” and “adults” and combinations between of them in Pub Med and Scopus database. The studies selected involved adult patients and included patients with co-morbidities, review articles and meta-analyses while the articles used were in English.

## Assessment of the Ability to Exercise

### Cardiopulmonary Exercise Testing

Cardiopulmonary Exercise Testing (CPET) and/or otherwise ergospirometry, is analogous to PSG in terms of systematically collecting information simultaneously. It is a non-invasive test that evaluates the function not only of the heart and lungs, but also of the whole body, both at rest but mainly during exercise. The test is performed on a cycle-ergometer and/or on treadmill and measurements are recorded from the cardiovascular, respiratory, circulatory and musculoskeletal systems. Specifically, within a strictly predetermined protocol with either a steady project increase in stages or with a continuous gradual project increase the ability of exercise and it is used in a wide range of clinical situations as it concerns all stages of each disease including diagnosis, severity assessment, disease progression, prognosis and response to treatment, to answer specific questions that arise after a basic clinical assessment (9).

### Contraindications to CPET

Contraindications to the assessment of the ability to exercise through the CPET (10) relate to the inability to perform a valid and satisfactory maximum/submaximal effort with an increased likelihood of the occurrence of an unpleasant incident during exercise (Supplementary Material).

### Six-Minute Walk Test

Six-minute walk test (6 MWT) is an additional assessment tools in patients with OSAS (11). 6 MWT is a non-invasive sub-maximal test it reviews the responses of exercise and evaluates the global and integrated responses of all the systems involved during exercise, including the pulmonary and cardiovascular systems, systemic circulation, peripheral circulation, blood, neuromuscular units, and muscle metabolism (12). The test is performed on 30-m on a flat hallway with hard surface, no exercise equipment and measures the distance that a patient can quickly walk in a period of 6 min (the 6 MWD). During 6 MWT are recorded the total of meters (m), arterial O_2_ saturation (SpO_2_), heart rate (HR), blood pressure (BP) and self-assessed lower extremity fatigue with dyspnea Borg Scale CR10 (13).

## Acute Responses to Exercise in OSAS Patients

### Cardiopulmonary Alterations

OSAS is a determinant of cardiovascular morbidity and mortality, with its most prominent cardiovascular complications involving drug-resistant hypertension, ischemic heart disease, cardiac arrhythmias and vascular stroke, while increased risk for sinus bradycardia, atrial and ventricular fibrillation, non-persistent ventricular tachycardia, activation of parasympathetic nervous system and bradyarrhythmias exist (14).OSAS patients showed altered hemodynamic response during exercise, while in the presence of comorbidities, the hemodynamic response to exercise is further impaired (15). Patients with OSAS experience a blunt chronotropic response to graded exercise due to variable dysregulation of cardiac β-receptors and/or baroreflex regulation site, resulting in impaired autonomic cardiovascular response (16, 17). Sleep apnea is conjoined with sleep fragmentation that creates a noxious environment prone to sympathetic excitation (18). As a result, alpha and beta – 2 receptors become desensitized, inducing vasoconstriction and endothelial dysfunction, as well as increasing heart rate (HR) and blood pressure (BP) (19, 20). This adrenergic blunted response becomes prominent during exercise, in which normally the operating point of baroreflex is set above mean arterial pressure, which is reset after exercise (21). In OSAS, the variability of baroreflex is attenuated and set centrally to higher operating point (22). OSAS patients who have undergone a CPET (Table 2) exhibit lower aerobic and anaerobic capacity compared to healthy individuals (27). According to Aron et al. (37), despite CPET being widely utilized for the evaluation and diagnosis of patients with coronary disease, studies have indicated significant differences in cardiorespiratory responses in OSAS patients. The authors observed that these patients demonstrate reduced ability to exercise and a reduced response of the heart rate (HR) to exercise compared to healthy individuals and concluded that these responses (low oxygen uptake and low heart rate) indicate a chronotropic disability. Moreover, OSAS patients have increased systolic and diastolic blood pressure during exercise and permanently elevated systolic blood pressure during the first minutes of the post-exercise recovery phase. These differences may be due to cardiac dysfunction, decreased muscle metabolism, chronic overactivation of the sympathetic nervous system (SNS) and endothelial dysfunction (17).

**Table 2:** Acute responses to exercise in patients with OSAS.

**References**	**Protocol**	**Results after exercise protocol**	
		**Increase**	**Decrease**
Grote et al. (23)	50 watts per 2 min^−1^	BP	HR
Tryfon et al. (24)	10, 15, or 20 watts per 1 min^−1^	BP	VO_2max_
Bonnani et al. (25)	3 min^−1^ submaximal test		VO_2max_, La
Oztruk et al. (26)	20 watts per 2 min^−1^		VO_2max_
Lin et al. (27)	1 min on 100 kpm		VO_2max_, anaerobic threshold
Kaleth et al. (28)	15 watts per 1 min^−1^		VO_2max_, HR, SBP
Vanhecke et al. (29)	Bruce test	BP	VO_2max_, HR
Ucok et al. (30)	Wingate test	% body fat	VO_2max_
Cintra et al. (31)	CPET maximal test	BP, LV	HDL
Rizzi et al. (32)	10–15 watts per 1 min^−1^	DBP	VO_2max_
Stavrou et al. (33)	15–20 watts per 1 min^−1^		VO_2max_, V_E_/MVV, VO_2_/HR
Stavrou et al. (34)	15–20 watts per 1 min^−1^	P_ET_CO_2_, BP	
Stavrou et al. (35)	15–20 watts per 1 min^−1^	Leg Fatigue	VO_2max_, HR
Stavrou et al. (36)	6 MWT	Dyspnea, Oxidative stress	Distance, HR

*CPET, cardiopulmonary exercise test; HDL, high-density lipoprotein; La, lactate acid; LV, left ventricular; MVV, maximum volunteer ventilation; P_ET_CO_2_, end-tidal carbon dioxide pressure, V_E_, ventilation; VO_2max_, maximal oxygen uptake; W, watts; BP, blood pressure (systolic / diastolic); DBP, diastolic blood pressure; HR, heart rate; SBP, systolic blood pressure; LV, left ventricular; 6MWT, 6 minute walk test*.

Heart failure arises due to repeated hypoxia-re-oxygenation and results in instability of the Autonomic Nervous System (ANS) (38). ANS instability is associated with endothelial dysfunction, vasoconstriction induced by SNS and enhanced response of β-2 receptor (23). According to Mansukhani et al. (38), there are several mechanisms by which blood pressure changes can occur during exercise in patients with OSAS. Disordered breathing with recurrent hypoxia-re-oxygenation circles has an impact on blood pressure response associated with endothelial dysfunction and ANS instability, while during polysomnography (PSG) in OSAS patients, arrythmias are reported (38). Decreased exercise capacity may indicate early cardiovascular dysfunction in these patients (23), while other factors contributing to reduced exercise may include weakened muscles and/or metabolic disorders (25).

Patients with OSAS have reduced pulmonary ventilation activity (reduced ERV relate to the cross-sectional area of the pharyngeal airway which decreases as lung volume decreases from FRC to residual volume suggesting the contribution of lung volumes). It is related to the severity of AHI and desaturation during sleep, interpreting an increased airway resistance during sleep, which is not sufficiently related with body composition (17). In addition, regulation of breathing during sleep is principally under the control of the chemoreceptors. The ventilatory feedback control system of chemoreflex is based on fluctuations of PaO_2_, which are more prominent in OSAS patients, making it vulnerable to instability (17). OSAS consists of repetitive episodes of apneas and hypopneas which activate the circle of intermittent hypoxia—hypercapnia, resulting in increased end-tidal CO_2_ while bicarbonate buffer system will attempt compensation by generating bicarbonate ions in addition to hydrogen ions, resulting in metabolic acidosis and alkalosis (17, 36, 39). Moreover, in patients with OSAS were observed higher values in maximum inspiratory pressure, which is associated with the severity of AHI (11). Concomitant with training programs in athletes, the intermittent breath holding during hypoxia-re-oxygenation, in patients with OSAS increases the intrathoracic pressure with successive alteration in the transmural pressure of the cardiac cavities, resulting increased respiratory muscles strength (11).

### Insulin - Resistant Syndromes and Pro-inflammatory Proneness

It is well-known that OSAS is related to other, beyond cardiopulmonary comorbidities such as type II diabetes and metabolic syndrome (40), as well as pro – inflammatory susceptibility. As far as diabetes mellitus and metabolic syndrome in relation to OSAS are concerned, researches have attributed their correlation to sleep fragmentation and particularly to sympathetic excitation and hypoxemia (Stavrou et al., 2019) (41). Sympathetic activity halts insulin secretion in islet beta cells, leading to OSAS patients often displaying impaired insulin sensitivity and increased plasma glucose levels (42), while studies have shown a significant statistical relationship between the severity of OSAS and insulin resistance (43). Furthermore, pancreatic beta cells require high supply of oxygen to support insulin secretion, rendering them sensitive to hypoxia (44). Hence, hypoxemia induced by sleep apnea, paves a direct and plausible relationship with insulin—resistant syndromes. Exercise could reverse such detrimental effects, as it restores vascular function by increasing NO bioavailability and balancing autonomic function, while it increases insulin sensitivity (45).

Sleep restriction, based on laboratory studies, is associated with a pre-inflammatory condition, which includes increase in inflammatory cytokines such as interleukins, Tumor Necrosis Factor (TNF) and C-reactive protein (CRP), regardless of obesity (46). Particularly, patients with OSAS due to hypoxia during sleep may experience low-grade systemic inflammation, which in turn may contribute to the onset and/or acceleration of the process of a widely prevalent inflammatory disease, atherosclerosis (47). According to Ruchała et al. (48), neurosteroids are synthesized in nervous system from cholesterol, steroid precursors and sex steroids, circulating in the blood stream and indirectly modulate breathing through gamma-aminobutyric acid (GABA) or N-methyl-D-aspartate (NMDA) signaling pathways. Testosterone is secreted on sleep patterns in particular first REM phase and plasma prolactin (PRL) concentrations show a sleep-dependent pattern, with increased secretion during sleep, while sleep deprivation can lead to lower levels. In addition, CPAP therapy is associated with a significant regulation of hormones serum level such as follicle-stimulating hormone (FSH), luteinizing hormone (LH), PRL, and testosterone. Moreover, Steiropoulos et al. (49) showed that in OSAS patients the night-time hypoxia can affect fasting insulin levels, even in non-diabetic OSAS patients, both a long-term Continuous Positive Airway Pressure (CPAP) treatment and short-term exercise without CPAP treatment (Stavrou et al., 2019) can significantly reduce HbA1c levels. Finally, OSAS patients present low vitamin-D levels. The low vitamin-D levels have been associated with multiple cardiovascular disorders, nervous system disorders (multiple sclerosis, amyotrophic lateral sclerosis, Parkinson's, and Alzheimer's), while CPAP treatment may increase vitamin-D levels OSAS patients (50, 51).

Therefore, exercise is expected to have an anti—inflammatory impact. However, only one study by Cavagnolli et al. (52), aimed to distinguish the anti - inflammatory effect of exercise in OSAS from its comorbidities and found C-RP was not significantly different between control and non-obese OSAS group. In a recent clinical trial by Jurado-García et al. (53), demonstrated that metabolic profile of obese OSAS patients improved after low—intensity exercise. Therefore, it becomes apparent that exercise acts indirectly through obesity in the amelioration of the systemic inflammatory environment. However, more studies are required to replicate the results.

### Oxidative Stress

Oxidative stress is a phenomenon caused by an imbalance between production and accumulation of Reactive Oxygen Species (ROS) or Reactive Oxygen Molecules (ROM) in cells and tissues and the ability of a biological system to detoxify these reactive products. ROS can play, and in fact they do it, several physiological roles (i.e., cell signaling), and they are normally generated as by-products of oxygen metabolism; despite this, environmental stressors (i.e., UV, ionizing radiations, pollutants, and heavy metals) and xenobiotics (i.e., antiblastic drugs) contribute to greatly increase ROS production, therefore causing the imbalance that leads to cell and tissue damage (oxidative stress) (54). Antioxidants are molecules that can donate an electron to a free radical without making themselves unstable, as this causes the free radical to stabilize and become less reactive. Several antioxidants have been exploited in recent years for their actual or supposed beneficial effect against oxidative stress. While we tend to describe oxidative stress as harmful for human body, it could as well be exploited as a therapeutic approach to treat clinical conditions (55).

It is a fact that oxidative stress has been associated with increased values in various diseases and therefore in diseases of the respiratory system (56). Thus, we can conclude that oxidative stress is also directly related to sleep disorders, and especially with obstructive sleep apnea according to studies. Obstructive sleep apnea syndrome can cause free oxygen radicals to be produced, due to the hypoxia/reoxygenation phenomenon, as reoxygenation can causes the production of these reactive oxygen species. Patients with severe OSAS have reduced values of antioxidant capacity, while antioxidant capacity is an index of excessive oxidative stress (57). OSAS itself can increase significantly the values of oxidative stress, given the fact that its patients have no other comorbidities or factors (58). In addition, patients with severe obstructive sleep apnea syndrome who presented increased oxidative stress, reduced the levels of oxidative stress after nasal CPAP treatment (59), but the antioxidant defense was not affected (60), while the values of ROMs in blood samples was associated with the severity of OSAS (61). It is worth emphasizing that sleep-disordered breathing has been recognized as a common, often unrecognized, comorbidity in patients with heart failure that is associated with increased mortality. Intermittent hypoxia in patients with sleep-disordered breathing could resemble ischemia–reperfusion injury, resulting in reactive oxygen species (ROS) generation during the reoxygenation period. Thus, sleep-disordered breathing is independently associated with enhanced oxidative stress in patients with heart failure (62).

Exercise itself is, also, linked to oxidative stress. Although acute exercise elevates ROS, systematic training prompts the organism to adapt to repetitive stimuli by increasing mitochondria biogenesis and antioxidant capacity (63). Hence, exercise brings additional benefits to OSAS patients, making a training program essential to supplement disease management.

## Mental Function Alterations

### Fatigue

Sleep disturbances prompt to an underestimated notion of one's exercise capabilities. Patients with OSAS demonstrate leg fatigue during mild exercise and/or physical activity, resulting in early cessation due to intolerance to exercise (64). According to Vanuxem et al. (65), leg fatigue reflects an impairment of muscle metabolism due to decreased peripheral O_2_ uptake, increased maximal lactate concentration and delayed lactate elimination in exercising muscles, resulting the occurrence of mitochondrial abnormalities in skeletal myofibres and the increased production of reactive O_2_ species exhibited in the neutrophils (25). In addition, the overestimation of the perceived sense of the leg fatigue is associated with cognitive decline, particularly with distinctive domains, in the framework of apneic episodes in OSAS patients (66). Such symptoms seem to promote a rather sedentary lifestyle, but they tend to ameliorate after treatment for OSAS (67).

Sleep disturbances influence acutely athletic performance. Chase et al. demonstrated that a single night of sleep restriction had a significant negative impact on athletic performance the following morning (68), while Rae et al. showed that even recovery from exercise was diminished after a single night of sleep deprivation (69). Concomitantly, two - night sleep deprivation affects executive function, as it causes central fatigue, signifying fewer high threshold motor units that can be recruited and, therefore, fewer muscle fibers will be activated to produce work (70).

### Cognition

OSAS as well as the severity of the syndrome can cause mental, cognitive and executive dysfunction, inability to concentrate, memory impairment and reduced activation of areas of the brain associated with knowledge. Some epidemiological studies have suggested a pathophysiological link between OSAS and Alzheimer's disease, which remains to be elucidated (5). According to Vanek et al. (71) attention, working memory, episodic memory, and executive functions are decreased in OSAS, due to different regions of the brain involved in cognition processes such as frontal cortex and hippocampus. Cerebral perfusion is altered during obstructive episodes, predisposing several brain areas relevant to cognitive performance to hypoxia (72). Nevertheless, the brain areas affected by apneic episodes have not been extrapolated to phenotypes of cognitive impairment, yet. Poor sleep quality is related to lower reaction time, after exhaustive exercise, in athletes due to compromised transmission velocity of neuronal impulses from the brain to working muscles (73). Perceptual ability (e.g., motor coordination), as well as attention and memory consolidation are hindered by acutely restricted sleep (36).

Exercise, particularly aerobic, along with a healthy and balanced diet, have been strongly linked to enhancing cognitive skills, as it increases cerebral perfusion (74). Executive function (75) and memory consolidation (76) have been indicated as examples of cognitive skills honed by exercise. However, further studies remain to fully elucidate the exact pathogenetic relationship of OSAS and cognitive impairment, especially in the context of exercise.

#### Anxiety and Mood Disorders

Several studies have adequately associated OSAS with mood and anxiety disorders (17). Sleep deprivation (chronic and/or acute) has a negative impact on cognitive performance, such as increased general anxiety, anxiety for failure, memory impairment, reduced concentration, and dysfunctional affective regulation (77, 78). According to Daabis and Gharraf (79), anxiety coexisting with depression in patients with OSAS and no CPAP treatment is associated with the general population, while depressive symptoms are highly prevalent in patients with moderate to severe OSAS and high BMI (80). Moreover, depression relate to functional decrease of serotoninergic neurotransmission, responsible for the alterations in sleep (79). Serotonin delivery to upper airway dilatator motor neurons reduce in dependency of the vigilance state, and lead to reductions in dilator muscle activity specifically during sleep (81).

## Chronic Responses to Exercise

Physical activity is considered to be one of the greatest lifestyle behaviors promoting health and is closely related to sleep quality, while there is a two-way relationship between sleep quality and physical activity (82). The low levels of physical activity in patients with OSAS, are due to early fatigue, daytime drowsiness and increased BMI. Pulmonary rehabilitation program (Table 3), with exercise being the main feature, in patients with OSAS show results that are associated with reduced AHI and ESS (91), while, at the same time, exercise has been shown to reduce the severity of other disorders or/and other OSAS related diseases, such as diabetes mellitus, cardiovascular disease, hypertension and obesity. The way exercise reduces the symptoms of OSAS is not fully understood, but studies have shown that the effect of exercise in patients with OSAS is not related to weight loss or BMI, but is possibly related to other mechanisms not yet understood (92). One possible explanation given, for exercise to reduce mild to severe AHI, focuses on the comorbidity of obesity and the obesity - hypoventilation syndrome, due to increased adipose tissue to the upper respiratory tract, leading to increased number of events of sleep apnea and/or hypopnea (93).

**Table 3:** Chronic responses to exercise in patients with OSAS.

**References**	**Protocol**	**Results after exercise intervention period**	
		**Increase**	**Decrease**
Norman et al. (83)	6 months (3 sessions/week) aerobic PA >3 METs + resistance exercise training + dietary consultation	VO_2max_, profile of mood states scores	AHI, Body fat, BP, ESS, Fatigue
Hambrecht et al. (84)	4 weeks, 3 times daily for 10 min on row ergometer and 3 times daily for 10 min on bicycle ergometer (warm-up 5 min, warm-down: 5 min). Workload exercise, so that did not experience chest pain and any signs of ischemia in the ECG	Vessel diameter, mean peak blood flow velocity, endothelium-dependent vasodilatation in LIMA	
Barnes et al. (85)	16 weeks aerobic exercise, resistance training, diet program (follow-up at 12 months).	VO_2max_, strength, quality of life	AHI, Body fat, HR in maximal effort, ESS, Cardiometabolic indexes
Kline et al. (86)	12 weeks (4 sessions/week), 150 min/week aerobic exercise on 60% of HRR and resistance exercises (4 sessions/week), 2 sets, 10–12 rep	Daily unsupervised activity, Sleep quality (PSQI)	AHI, Body fat
Yang et al. (87)	12 weeks (3 sessions/week), 30 min aerobic exercise on AT	SpO_2_	AHI, BMI, HRR
Servantes et al. (88)	3/week for 3 months, 30–70 years, NYHA class II to III, AHI ≥ 5/h with symptoms or AHI ≥15/h, randomized four groups (A: control, B: exercise, C: CPAP, D: exercise + CPAP) B + C group: warm-up: 10-min, aerobic training: ±10 bpm form HR_AT_ of CPET (treadmill and cycloergometer; 1 month 30 min, 2 months 45-min) and strength training (three exercises for upper limbs and four exercises for lower limbs, 1-min rest period, free weights) 50–60% of 1 RM	VO_2max_	AHI, ESS, Quality of life
Yilmaz et al. (89)	12 weeks (5 sessions/week), 60-min Tai-Chi & Qigong (3 sessions/week in rehabilitation center and 2 sessions/week self-selected)	SpO_2_, Sleep quality (PSQI)	AHI, ESS
Stavrou et al. (39)	4 weeks (3 sessions/week), 4 set for 5 min with 1 min rest on 70% of VO_2max_	VO_2max_	AHI, BP, HbA1-c, LDL
Berger et al. (90)	9 months 3 h/week supervised community physical activity program (Nordic walking, gymnastics, and aqua gym), 40–80 years, 15–30 AHI/h, warm-up 10-min, 40-min combined resistance and aerobic exercises at the anaerobic threshold, and cooldown 10-min stretches	VO_2max_	AHI, Nighttime HRV, Cardiovascular risk

*1 RM, one-repetition maximum; AHI, apnea-hypopnea index; AT, anaerobic threshold; avergSpO_2_, mean oxygen saturation during polysomnography study; BMI, body mass index; BP, blood pressure; CPAP, Continuous positive airway pressure; CPET, cardiopulmonary exercise test; ECG, Electrocardiography; ESS, Epworth sleepiness scale; HbA1-c, hemoglobin A1c; HRR, heart rate reserve; LDL, low-density lipoprotein; LIMA, left internal mammary artery; NYHA, New York Heart Association; PA, physical activity; PSQI, Pittsburgh Sleep Quality Index; VO_2max_, oxygen uptake in maximal effort*.

In addition, physical activity in patients with OSAS has been observed to present a protective role in the course of the disease, without, however, representing a reduction in maximal oxygen uptake, an indicator associated with health status (35). A supervised physical activity may prevent a decline in nighttime cardiac autonomic function (CAF) and may be cardioprotective in OSAS patients with bradycardia, CAF preservation, and increase O_2_ uptake in maximal effort (90). According to Servantes et al. (88), both exercise and CPAP treatment improved subjective excessive daytime sleepiness, quality of life, and the NYHA functional class distribution. Furthermore, peak O_2_ consumption, as health indicator, can be improved only with exercise (strength and endurance) while exercise can reduce AHI and have important implications in the management of patients with HF and OSAS (88).

Exercise can lead to a reduction in body weight and BMI and therefore a reduction in adipose tissue in the upper tract and in the pharyngeal region (94), while at the same time exercise improves the levels of physical activity and in combination with application of CPAP machine, there is additional improvement in patients' sleep symptoms and quality of life. The increase of physical activity improves patients' health indicators and can significantly reduce the cardiovascular risk factors associated with OSAS (39). Finally, exercise in patients with OSAS, improves quality of life and mood, reduces levels of anger, depression, bodily pain, and total mood disturbances, and increased participation in social activities (95). Exercise contributes positive biological and psychological effects that affect the brain and the cognitive functioning and promote a condition of well-being, while triggers potent neuroplastic phenomena, partly mediated by epigenetic mechanisms (96).

## Conclusion

Patients with OSAS exhibit systematic detrimental effects, with tremendous impact on quality of life, if left untreated. Cardiopulmonary implications, as well as endocrine dysregulation and cognitive impairment consist of the main consequences. Patients with OSAS exhibit acute exercise responses related to OSAS such as reduced ability to exercise, lower aerobic and anaerobic capacity compared to healthy individuals. They also exhibit chronic responses such as prolonged physical inactivity. As a result, a specific exercise program targeting patients with OSAS is described in detail, in order to ameliorate the systematic consequences of OSAS, as well as to propose the prescription of an exercise program as a supplementary therapeutic intervention for these patients.

## Author Contributions

VS and KG conceived of the presented idea and designed the study. VS, KT, GV, EP, KA, and SB contributed to the writing the paper. VS designed the figures and tables. ZD and KG supervised the study. All authors contributed to the article and approved the submitted version.

## Conflict of Interest

The authors declare that the research was conducted in the absence of any commercial or financial relationships that could be construed as a potential conflict of interest.

## Publisher's Note

All claims expressed in this article are solely those of the authors and do not necessarily represent those of their affiliated organizations, or those of the publisher, the editors and the reviewers. Any product that may be evaluated in this article, or claim that may be made by its manufacturer, is not guaranteed or endorsed by the publisher.

## References

[1] American Thoracic Society. Standards and indications for cardiopulmonary sleep studies in children. Am J Respir Crit Care Med. (1996) 153:855–78. 10.1164/ajrccm.153.2.8564147 8564147

[2] Franklin KA Lindberg E. Obstructive sleep apnea is a common disorder in the population-a review on the epidemiology of sleep apnea. J Thorac Dis. (2015) 7:1311–22. 10.3978/j.issn.2072-1439.2015.06.11 26380759 PMC4561280

[3] Cai A Zhou Y Zhang J Zhong Q Wang R Wang L. Epidemiological characteristics and gender-specific differences of obstructive sleep apnea in a Chinese hypertensive population: a cross-sectional study. BMC Cardiovasc Disord. (2017) 17:8. 10.1186/s12872-016-0447-4 28056799 PMC5217623

[4] Kushida AC Littner RM Morgenthaler T Alessi AC Bailey D Coleman J. Practice Parameters for the Indications for Polysomnography and Related Procedures: An Update for 2005. Sleep (2005) 28:499–523. 10.1093/sleep/28.4.499 16171294

[5] Valgimigli M Ceconi C Malagutti P Merli E Soukhomovskaia O Francolini G. Tumor necrosis factor-alpha receptor 1 is a major predictor of mortality and new-onset heart failure in patients with acute myocardial infarction: the cytokine-activation and long-term prognosis in myocardial infarction (C-ALPHA) study. Circulation. (2005) 111:863–70. 10.1161/01.CIR.0000155614.35441.69 15699251

[6] Mattis J Sehgal A. Circadian rhythms, sleep, and disorders of aging. Trends Endocrinol Metab. (2016) 27:192–203. 10.1016/j.tem.2016.02.003 26947521 PMC4808513

[7] Simpson L McArdle N Eastwood PR Ward KL Cooper MN Wilson AC. Physical inactivity is associated with moderate-severe obstructive sleep apnea. J Clin Sleep Med. (2015) 11:1091–9. 10.5664/jcsm.5078 26285117 PMC4582050

[8] Iftikhar IH Kline CE Youngstedt SD. Effects of exercise training on sleep apnea: a meta-analysis. Lung. (2014) 192:175–84. 10.1007/s00408-013-9511-3 24077936 PMC4216726

[9] Stavrou VT Astara K Tourlakopoulos KN Daniil Z Gourgoulianis KI Kalabakas K. Sleep quality's effect on vigilance and perceptual ability in adolescent and adult athletes. J Sports Med. (2021) 2021:9. 10.1155/2021/5585573 33937414 PMC8055422

[10] Levett DZH Jack S Swart M Carlisle J Wilson J Snowden C. Perioperative cardiopulmonary exercise testing (CPET): consensus clinical guidelines on indications, organization, conduct, and physiological interpretation. Br J Anaesth. (2018) 120:484–500. 10.1016/j.bja.2017.10.020 29452805

[11] Stavrou VT Astara K Karetsi E Daniil Z Gourgoulianis KI. Respiratory muscle strength as an indicator of the severity of apnea hypopnea index: stepping towards the distinction between sleep apnea and breath holding. Cureus. (2021) 13:e14015. 10.7759/cureus.14015 33889460 PMC8056360

[12] ATS Committee on Proficiency Standards for Clinical Pulmonary Function Laboratories. ATS statement: guidelines for the six-minute walk test. Am J Respir Crit Care Med. (2002) 166:111–7. 10.1164/ajrccm.166.1.at1102 12091180

[13] Borg E Borg G Larsson K Letzter M Sundblad BM. An index for breathlessness and leg fatigue. Scand J Med Sci Sports. (2010) 20:644–50. 10.1111/j.1600-0838.2009.00985.x 19602182

[14] Natsios G Pastaka C Vavougios G Zarogiannis SG Tsolaki V Dimoulis A. Age, body mass index, and daytime and nocturnal hypoxia as predictors of hypertension in patients with obstructive sleep apnea. J Clin Hypertens. (2016) 18:146–52. 10.1111/jch.12645 26252911 PMC8032090

[15] Abdeyrim A Zhang Y Li N Zhao M Wang Y Yao X. Impact of obstructive sleep apnea on lung volumes and mechanical properties of the respiratory system in overweight and obese individuals. BMC Pulm Med. (2015) 15:76. 10.1186/s12890-015-0063-6 26209328 PMC4513967

[16] Somers VK Dyken ME Mark AL Abboud FM. Sympathetic-nerve activity during sleep in normal subjects. N Engl J Med. (1993) 328:303–7. 10.1056/NEJM199302043280502 8419815

[17] Stavrou V Bardaka F Karetsi E Daniil Z Gourgoulianis KI. Brief review: ergospirometry in patients with obstructive sleep apnea syndrome. J Clin Med. (2018) 7:191. 10.3390/jcm7080191 30065219 PMC6111535

[18] Goff EA Nicholas CL Simonds AK Trinder J Morrell MJ. Differential effects of waking from non-rapid eye movement versus rapid eye movement sleep on cardiovascular activity. J Sleep Res. (2010) 19(1 Pt 2):201–6. 10.1111/j.1365-2869.2009.00783.x 19878448

[19] Grote L Kraiczi H Hedner J. Reduced alpha- and beta(2)-adrenergic vascular response in patients with obstructive sleep apnea. Am J Respir Crit Care Med. (2000) 162 (4 Pt 1):1480–7. 10.1164/ajrccm.162.4.9912028 11029365

[20] Mills PJ Dimsdale JE Coy TV Ancoli-Israel S Clausen JL Nelesen RA. Beta 2-adrenergic receptor characteristics in sleep apnea patients. Sleep. (1995) 18:39–42. 10.1093/sleep/18.1.39 7761741

[21] DiCarlo SE Bishop VS. Central baroreflex resetting as a means of increasing and decreasing sympathetic outflow and arterial pressure. Ann N Y Acad Sci. (2001) 940:324–37. 10.1111/j.1749-6632.2001.tb03688.x 11458690

[22] Cooper VL Elliott MW Pearson SB Taylor CM Mohammed MM Hainsworth R. Daytime variability of baroreflex function in patients with obstructive sleep apnoea: implications for hypertension. Exp Physiol. (2007) 92:391–8. 10.1113/expphysiol.2006.035584 17204492

[23] Grote L Hedner J Peter H. The heart rate response to exercise is blunted in patients with sleep-related breathing disorder. Cardiology. (2004) 102:93–9. 10.1159/000077911 15103179

[24] Tryfon S Stanopoulos I Dascalopoulou E Argyropoulou P Bouros D Mavrofridis E. Sleep apnea syndrome and diastolic blood pressure elevationduring exercise. Respiration. (2004) 71:499–504. 10.1159/000080635 15467328

[25] Bonanni E Pasquali L Manca ML Maestri M Prontera C Fabbrini M. Lactate production and catecholamine profile during aerobic exercise in normotensive OSAS patients. Sleep Med. (2004) 5:137–45. 10.1016/j.sleep.2003.08.009 15033133

[26] Ozturk LM Metin G Cuhadaroglu C Utkusavas A Tutluoglu B. Cardiopulmonary responses to exercise in moderate- to-severe obstructive sleep apnea. Tuberk Toraks. (2005) 53:10–9. 15765282

[27] Lin CC Hsieh WY Chou CS Liaw SF. Cardiopulmonary exercise testing in obstructive sleep apnea syndrome. Respir Physiol Neurobiol. (2006) 150:27–34. 10.1016/j.resp.2005.01.008 16448931

[28] Kaleth AS Chittenden TW Hawkins BJ Hargens TA Guill SG Zedalis D. Unique cardiopulmonary exercise test responses in overweight middle-aged adults with obstructive sleep apnea. Sleep Med. (2007) 8:160–8. 10.1016/j.sleep.2006.08.005 17275399

[29] Vanhecke T Franklin B Zalesin K Sangal B deJong A Agrawal V. Cardiorespiratory fitness and obstructive sleep apnea syndrome in morbidly obese patients. Chest. (2008) 134:539–45. 10.1378/chest.08-0567 18779193

[30] Ucok K Aycicek A Sezer M Genc A Akkaya M Caglar V. Aerobic and anaeroc exercise capacities in obstructive sleep apnea and associations with subcutaneous fat distributions. Lung. (2009) 187:29–36. 10.1007/s00408-008-9128-0 19023624

[31] Cintra FD Tufik S Paola AD Feres MC Melo-Fujita L Oliveira W Rizzi C. Cardiovascular profile in patients with obstructive sleep apnea. Arq Bras Cardiol. (2011) 96:293–9. 10.1590/S0066-782X2011005000030 21437515

[32] Rizzi CF Cintra F Mello-Fujita L Rios LF Mendonca ET Feres MC. Does obstructive sleep apnea impair the cardiopulmonary response to exercise? Sleep. (2013) 36:547–53. 10.5665/sleep.2542 23565000 PMC3612253

[33] Stavrou V Vavougios G Pastaka CH Daniil Z Gourgoulianis K Karetsi E. The cardiopulmonary exercise testing as a novel predictive tool of sleep apnea syndrome. ERS Int Congr. (2015) 46(Suppl. 59):PA2321. 10.1183/13993003.congress-2015.PA2321

[34] Stavrou V Boutou AK Vavougios GD Pastaka C Gourgoulianis KI Koutedakis Y. The use of cardiopulmonary exercise testing in identifying the presence of obstructive sleep apnea syndrome in patients with compatible symptomatology. Respir Physiol Neurobiol. (2019) 262:26–31. 10.1016/j.resp.2019.01.010 30684645

[35] Stavrou V Bardaka F Karetsi E Seitanidis G Daniil Z Gourgoulianis KI. The effect of physical strain on breeders patients with obstructive sleep apnea syndrome. Respir Physiol Neurobiol. (2019) 260:137–9. 10.1016/j.resp.2018.11.009 30472194

[36] Stavrou VT Vavougios GD Astara K Siachpazidou DI Papayianni E Gourgoulianis KI. The 6-minute walk test and anthropometric characteristics as assessment tools in patients with Obstructive Sleep Apnea Syndrome. A preliminary report during the pandemic. J Personal Med. (2021) 11:563. 10.3390/jpm11060563 34208496 PMC8234449

[37] Aron A Zedalis D Gregg JM Gwazdauskas FC Herbert WG. Potential clinical use of cardiopulmonary exercise testing in obstructive sleep apnea hypopnea syndrome. Int J Cardiol. (2009) 132:176–86. 10.1016/j.ijcard.2008.11.014 19042045

[38] Mansukhani MP Allison TG Lopez-Jimenez F Somers VK Caples SM. Functional aerobic capacity in patients with sleep-disordered breathing. Am J Cardiol. (2013) 111:1650–4. 10.1016/j.amjcard.2013.02.008 23578347 PMC4014074

[39] Stavrou V Karetsi E Daniil Z Gourgoulianis KI. 4 weeks exercise in obstructive sleep apnea syndrome patient with type 2 diabetes mellitus and without continuous positive airway pressure treatment: a case report. Sleep Med Res. (2019) 10:1–5. 10.17241/smr.2019.00374

[40] Heinzer R Vat S Marques-Vidal P Marti-Soler H Andries D Tobback N. Prevalence of sleep-disordered breathing in the general population: the HypnoLaus study. Lancet Respir Med. (2015) 3:310–8. 10.1016/S2213-2600(15)00043-0 25682233 PMC4404207

[41] Murphy AM Thomas A Crinion SJ Kent BD Tambuwala MM Fabre A. Intermittent hypoxia in obstructive sleep apnoea mediates insulin resistance through adipose tissue inflammation. Eur Respir J. (2017) 49:1601731. 10.1183/13993003.01731-2016 28424360

[42] Ryan S. Adipose tissue inflammation by intermittent hypoxia: mechanistic link between obstructive sleep apnoea and metabolic dysfunction. J Physiol. (2017) 595:2423–30. 10.1113/JP273312 27901270 PMC5390885

[43] Iftikhar IH Hoyos CM Phillips CL. Meta-analyses of the association of sleep apnea with insulin resistance, and the effects of CPAP on HOMA-IR, adiponectin, and visceral adipose fat. J Clin Sleep Med. (2015) 11:475–85. 10.5664/jcsm.4610 25700870 PMC4365462

[44] Olsson R Olerud J Pettersson U Carlsson PO. Increased numbers of low-oxygenated pancreatic islets after intraportal islet transplantation. Diabetes. (2011) 60:2350–3. 10.2337/db09-0490 21788575 PMC3161309

[45] Garber CE Blissmer B Deschenes MR Franklin BA Lamonte MJ Lee IM. American College of Sports Medicine position stand. Quantity and quality of exercise for developing and maintaining cardiorespiratory, musculoskeletal, and neuromotor fitness in apparently healthy adults: guidance for prescribing exercise. Med Sci Sports Exerc. (2011) 43:1334–59. 10.1249/MSS.0b013e318213fefb 21694556

[46] Perelis M Ramsey KM Marcheva B Bass J. Circadian transcription from beta cell function to diabetes path-ophysiology. J Biol Rhythms. (2016) 31:323–36. 10.1177/0748730416656949 27440914 PMC8985168

[47] Jordan A McSharry D Malhotra A. Adult obstructive sleep apnoea. Lancet. (2014) 22:736–47. 10.1016/S0140-6736(13)60734-5 23910433 PMC3909558

[48] Ruchała M Bromińska B Cyrańska-Chyrek E Kuznar-Kamińska B Kostrzewska M Batura-Gabryel H. Obstructive sleep apnea and hormones - a novel insight. Arch Med Sci. (2017) 13:875–84. 10.5114/aoms.2016.61499 28721156 PMC5507108

[49] Steiropoulos P Papanas N Nena E Tsara V Fitili C Tzouvelekis A. Markers of glycemic control and insulin resistance in non-diabetic patients with obstructive sleep apnea hypopnea syndrome: does adherence to CPAP treatment improve glycemic control? Sleep Med. (2009) 10:887–91. 10.1016/j.sleep.2008.10.004 19231280

[50] Siachpazidou DI Stavrou V Zouridis S Gogou E Economou NT Pastaka C. 25-hydroxyvitamin D levels in patients with obstructive sleep apnea and continuous positive airway pressure treatment: a brief review. Sleep Sci. (2020) 13:78–83. 10.1007/s11325-020-02146-6 32670496 PMC7347362

[51] Siachpazidou DI Stavrou VT Astara K Pastaka C Gogou E Hatzoglou C. Alzheimer disease in patients with obstructive sleep apnea syndrome. Tanaffos. (2020) 19:176–85. 33815537 PMC8008406

[52] Cavagnolli DA Esteves AM Ackel-D'Elia C Maeda MY de Faria AP Tufik S. Aerobic exercise does not change C-reactive protein levels in non-obese patients with obstructive sleep apnoea. Eur J Sport Sci. (2014) 14(Suppl. 1):S142–7. 10.1080/17461391.2012.663412 24444198

[53] Jurado-García A Molina-Recio G Feu-Collado N Palomares-Muriana A Gómez-González AM Márquez-Pérez FL. Effect of a graduated walking program on the severity of obstructive sleep apnea syndrome. A randomized clinical trial. Int J Environ Res Public Health. (2020) 17:6334. 10.3390/ijerph17176334 32878112 PMC7503647

[54] Pizzino G Irrera N Cucinotta M Pallio G Mannino F Arcoraci V. Oxidative stress: harms and benefits for human health. Oxid Med Cell Longev. (2017) 2017:8416763. 10.1155/2017/8416763 28819546 PMC5551541

[55] Betteridge DJ. What is oxidative stress? Metabolism. (2000) 49(2 Suppl. 1):3–8. 10.1016/S0026-0495(00)80077-3 10693912

[56] Papageorgiou E Kostikas K Kiropoulos T Karetsi E Mpatavanis G Gourgoulianis KI. Increased oxidative stress in exudative pleural effusions: a new marker for the differentiation between exudates and transudates? Chest. (2005) 128:3291–7. 10.1378/chest.128.5.3291 16304274

[57] Christou K Markoulis N Moulas AN Pastaka C Gourgoulianis KI. Reactive oxygen metabolites (ROMs) as an index of oxidative stress in obstructive sleep apnea patients. Sleep Breath. (2003) 7:105–10. 10.1007/s11325-003-0105-9 14569521

[58] Ntalapascha M Makris D Kyparos A Tsilioni I Kostikas K Gourgoulianis K. Oxidative stress in patients with obstructive sleep apnea syndrome. Sleep Breath. (2013) 17:549–55. 10.1007/s11325-012-0718-y 22610662

[59] Christou K Kostikas K Pastaka C Tanou K Antoniadou I Gourgoulianis KI. Nasal continuous positive airway pressure treatment reduces systemic oxidative stress in patients with severe obstructive sleep apnea syndrome. Sleep Med. (2009) 10:87–94. 10.1016/j.sleep.2007.10.011 18077211

[60] Alzoghaibi MA Bahammam AS. The effect of one night of continuous positive airway pressure therapy on oxidative stress and antioxidant defense in hypertensive patients with severe obstructive sleep apnea. Sleep Breath. (2012) 16:499–504. 10.1007/s11325-011-0531-z 21567336

[61] Christou K Moulas AN Pastaka C Gourgoulianis KI. Antioxidant capacity in obstructive sleep apnea patients. Sleep Med. (2003) 4:225–8. 10.1016/S1389-9457(02)00253-8 14592326

[62] Kondo M Kohno T Kohsaka S Fukuoka R Shiraishi Y Sawano M. Enhanced oxidative stress is associated with sleep-disordered breathing and obesity in patients with heart failure. Int J Cardiol. (2016) 15:133–5. 10.1016/j.ijcard.2016.02.042 26889597

[63] Kawamura T Muraoka I. Exercise-induced oxidative stress and the effects of antioxidant intake from a physiological viewpoint. Antioxidants. (2018) 7:119. 10.3390/antiox7090119 30189660 PMC6162669

[64] Aguillard RN Riedel BW Lichstein KL Grieve FG Johnson CT Noe SL. Daytime functioning in obstructive sleep apnea patients: exercise tolerance, subjective fatigue, and sleepiness. Appl Psychophysiol Biofeedback. (1998) 23:207–17. 10.1023/A:1022257514209 10457812

[65] Vanuxem D Badier M Guillot C Delpierre S Jahjah F Vanuxem P. Impairment of muscle energy metabolism in patients with sleep apnoea syndrome. Respir Med. (1997) 91:551–7. 10.1016/S0954-6111(97)90089-5 9415356

[66] Astara K Stavrou VT Vavougios GD Siachpazidou DI Papayianni E Gourgoulianis KI. Fitness indicators and cognitive performance in patients with obstructive sleep apnea syndrome. A preliminary report. ERJ Open Res. (2021) 7(Suppl. 7):52. 10.1183/23120541.sleepandbreathing-2021.52 PMC8234449 34208496

[67] Chotinaiwattarakul W O'Brien LM Fan L Chervin RD. Fatigue, tiredness, and lack of energy improve with treatment for OSA. J Clin Sleep Med. (2009) 5:222–7. 10.5664/jcsm.27490 19960642 PMC2699166

[68] Chase JD Roberson PA Saunders MJ Hargens TA Womack CJ Luden ND. One night of sleep restriction following heavy exercise impairs 3-km cycling time-trial performance in the morning. Appl Physiol Nutr Metab. (2017) 42:909–15. 10.1139/apnm-2016-0698 28467857

[69] Rae DE Chin T Dikgomo K Hill L McKune AJ Kohn TA. One night of partial sleep deprivation impairs recovery from a single exercise training session. Eur J Appl Physiol. (2017) 117:699–712. 10.1007/s00421-017-3565-5 28247026

[70] Skurvydas A Kazlauskaite D Zlibinaite L Cekanauskaite A Valanciene D Karanauskiene D. Effects of two nights of sleep deprivation on executive function and central and peripheral fatigue during maximal voluntary contraction lasting 60s. Physiol Behav. (2021) 229:113226. 10.1016/j.physbeh.2020.113226 33122092

[71] Vanek J Prasko J Genzor S Ociskova M Kantor K Holubova M. Obstructive sleep apnea, depression and cognitive impairment. Sleep Med. (2020) 72:50–8. 10.1016/j.sleep.2020.03.017 32544796

[72] O'Donoghue FJ Wellard RM Rochford PD Dawson A Barnes M Ruehland WR. Magnetic resonance spectroscopy and neurocognitive dysfunction in obstructive sleep apnea before and after CPAP treatment. Sleep. (2012) 35:41–8. 10.5665/sleep.1582 22215917 PMC3242686

[73] Fullagar HH Skorski S Duffield R Hammes D Coutts AJ Meyer T. Sleep and athletic performance: the effects of sleep loss on exercise performance, and physiological and cognitive responses to exercise. Sports Med. (2015) 45:161–86. 10.1007/s40279-014-0260-0 25315456

[74] Gorelick PB Scuteri A Black SE Decarli C Greenberg SM Iadecola C. Vascular contributions to cognitive impairment and dementia: a statement for healthcare professionals from the american heart association/american stroke association. Stroke. (2011) 42:2672–713. 10.1161/STR.0b013e3182299496 21778438 PMC3778669

[75] Kleinloog JPD Mensink RP Ivanov D Adam JJ Uludag K Joris PJ. Aerobic exercise training improves cerebral blood flow and executive function: a randomized, controlled cross-over trial in sedentary older men. Front Aging Neurosci. (2019) 11:333. 10.3389/fnagi.2019.00333 31866855 PMC6904365

[76] Maass A Düzel S Goerke M Becke A Sobieray U Neumann K. Vascular hippocampal plasticity after aerobic exercise in older adults. Mol Psychiatry. (2015) 20:585–93. 10.1038/mp.2014.114 25311366

[77] Matsumoto Y Mishima K Satoh K Shimizu T Hishikawa Y. Physical activity increases the dissociation between subjective sleepiness and objective performance levels during extended wakefulness in human. Neurosci Lett. (2002) 326:133–6. 10.1016/S0304-3940(02)00335-X 12057846

[78] Kamphuis J Meerlo P Koolhaas JM Lancel M. Poor sleep as a potential causal factor in aggression and violence. Sleep Med. (2012) 13:327–34. 10.1016/j.sleep.2011.12.006 22305407

[79] Daabis R Gharraf H. Predictors of anxiety and depression in patients with obstructive sleep apnea. Egypt J Chest Dis Tuberculosis. (2012) 61:171–7. 10.1016/j.ejcdt.2012.10.03221484722

[80] Rezaeitalab F Moharrari F Saberi S Asadpour H Rezaeetalab F. The correlation of anxiety and depression with obstructive sleep apnea syndrome. J Res Med Sci. (2014) 19:205–10. 24949026 PMC4061640

[81] Veasey SC. Serotonin agonists and antagonists in obstructive sleep apnea: therapeutic potential Am. J Respir Med. (2003) 2:21–9. 10.1007/BF03256636 14720019

[82] Mendelson M Bailly S Marillier M Flore P Borel JC Vivodtzev I. Obstructive sleep apnea syndrome, objectively measured physical activity and exercise training interventions: a systematic review and meta-analysis. Front Neurol. (2018) 9:73. 10.3389/fneur.2018.00073 29520251 PMC5827163

[83] Norman JF Von Essen SG Fuchs RH McElligott M. Exercise training effect on obstructive sleep apnea syndrome. Sleep Res Online. (2000) 3:121–9. 11382910

[84] Hambrecht R Adams V Erbs S Linke A Kränkel N Shu Y. Regular physical activity improves endothelial function in patients with coronary artery disease by increasing phosphorylation of endothelial nitric oxide synthase. Circulation. (2003) 107:3152–8. 10.1161/01.CIR.0000074229.93804.5C 12810615

[85] Barnes M Goldsworthy UR Cary BA Hill CJ. A diet and exercise program to improve clinical outcomes in patients with obstructive sleep apnea–a feasibility study. J Clin Sleep Med. (2009) 5:409–15. 10.5664/jcsm.27594 19961023 PMC2762710

[86] Kline CE Crowley EP Ewing GB Burch JB Blair SN Durstine JL. The effect of exercise training on obstructive sleep apnea and sleep quality: a randomized controlled trial. Sleep. (2011) 34:1631–40. 10.5665/sleep.1422 22131599 PMC3208839

[87] Yang H Liu Y Zheng H Liu G Mei A. Effects of 12 weeks of regular aerobic exercises on autonomic nervous system in obstructive sleep apnea syndrome patients. Sleep Breath. (2018) 22:1189–95. 10.1007/s11325-018-1736-1 30328014

[88] Servantes DM Javaheri S Kravchychyn ACP Storti LJ Almeida DR de Mello MT. Effects of exercise training and CPAP in patients with Heart Failure and OSA: a preliminary study. Chest. (2018) 154:808–17. 10.1016/j.chest.2018.05.011 30213463

[89] Yilmaz GG Akkoyunlu ME Kikic L Algun C. The effect of T'ai Chi and Qigong training on patients with obstructive sleep apnea: a randomized controlled study. J Altern Complement Med. (2019) 25:317–25. 10.1089/acm.2018.0197 30427696

[90] Berger M Raffin J Pichot V Hupin D Garet M Labeix P. Effect of exercise training on heart rate variability in patients with obstructive sleep apnea: a randomized controlled trial. Scand J Med Sci Sports. (2019) 29:1254–62. 10.1111/sms.13447 31050034

[91] Aiello AE Feinstein L Dowd JB Pawelec G Derhovanessian E Galea S. Income and markers of immunological cellular aging. Psychosom Med. (2016) 78:657–66. 10.1097/PSY.0000000000000320 27187853 PMC4927391

[92] Awad KM Malhotra A Barnet JH Quan SF Peppard PE. Exercise is associated with a reduced incidence of sleep-disordered breathing. Am J Med. (2012) 125:485–90. 10.1016/j.amjmed.2011.11.025 22482846 PMC3339801

[93] Myers KA Mrkobrada M Simel DL. Does this patient have obstructive sleep apnea?: the rational clinical examination systematic review. JAMA. (2016) 310:731e741. 10.1001/jama.2013.276185 23989984

[94] Greenburg DL Lettieri CJ Eliasson AH. Effects of surgical weight loss on measures of obstructive sleep apnea: a meta-analysis. Am J Med. (2009) 122:535e542. 10.1016/j.amjmed.2008.10.037 19486716

[95] Butner KL Hargens TA Kaleth AS Miller LE Zedalis D Herbert WG. Association of obstructive sleep apnea severity with exercise capacity and health-related quality of life. N Am J Med Sci. (2013) 5:362–6. 10.4103/1947-2714.114168 23923110 PMC3731867

[96] Mandolesi L Polverino A Montuori S Foti F Ferraioli G Sorrentino P. Effects of physical exercise on cognitive functioning and wellbeing: biological and psychological benefits. Front Psychol. (2018) 27:509. 10.3389/fpsyg.2018.00509 29755380 PMC5934999

