# Supplementary material for: Obstructive Sleep Apnea Syndrome: The Effect of Acute and Chronic Responses of Exercise
Source: Front Med (Lausanne). 2021 Dec 24;8:806924. doi: 10.3389/fmed.2021.806924 (PMC8738168; doi:10.3389/fmed.2021.806924)
Supplement: Supplementary file 1 [file Data_Sheet_1.docx]

**Cardiopulmonary Exercise Testing**

Contraindications to CPET

The absolute contraindications type I are:

1. recent (3-5 days) acute myocardial infarction
2. unstable angina, uncontrolled arrhythmia that causes symptoms or hemodynamic instability
3. syncope
4. acute endocarditis
5. acute myocarditis or pericarditis
6. symptomatic severe aortic stenosis
7. uncontrolled heart failure
8. acute pulmonary embolism or pulmonary infarction and lower limb thrombosis

The absolute contraindications type II are:

1. suspected aneurysm split
2. uncontrolled asthma
3. pulmonary edema
4. arterial blood saturation ≤ 85% at rest
5. acute non-cardiorespiratory disturbance which may impair exercise ability (e.g., thyrotoxicosis, acute infection) and mental disorders that make patients uncapable of consenting and cooperating

Relative contraindications are:

1. left coronary artery stenosis
2. moderate and severe valvular stenosis
3. untreated arterial hypertension at rest (systolic blood pressure >200 mmHg, diastolic blood pressure >120 mmHg)
4. tachyarrhythmias or bradyarrhythmias
5. high-grade atrioventricular block (2^nd^>)
6. hypertrophic cardiomyopathy
7. severe pulmonary arterial hypertension
8. advanced or complicated pregnancy (advanced maternal or 3^rd^ trimester)
9. electrolyte imbalances and orthopedic disorders that limit the ability to exercise

Each CPET can be completed when the participant reached symptom-limited maximum exercise,

1. RER >1.10
2. HR ≥80% of predicted HRmax
3. plateau of oxygen consumption with increasing work load
4. Borg scale CR10 for leg fatigue and Dyspnea ≥5

Reasons for immediately stopping a CPET include the following:

1. chest pain
2. intolerable dyspnea
3. leg cramps
4. pale or ashen appearance
5. diaphoresis
6. adnormal ECG
7. reduced arterial pressure and/or HR with increasing work load and /or oxygen consumption
8. wish of participant
9. weakness to maintaining a steady rpm

**Six-minute walking test**

Reasons for immediately stopping a 6MWT include the following:

1. chest pain
2. intolerable dyspnea
3. leg cramps
4. staggering
5. diaphoresis
6. pale or ashen appearance
